# Leaner, Healthier, Happier Together––A Family-Centred Approach to Weight Loss with the Overweight Dog and Her Caregivers

**DOI:** 10.3390/vetsci4030041

**Published:** 2017-08-22

**Authors:** Alessia Candellone, David Morgan, Simona Buttignol, Giorgia Meineri

**Affiliations:** 1 Department of Veterinary Science, University of Turin, 10095 Grugliasco, Italy; giorgia.meineri@unito.it; 2 Spectrum Brands Schweiz GmbH, 8305 Dietlikon, Switzerland; David.Morgan@eu.spectrumbrands.com; 3 Ambulatorio Veterinario Associato dott.sse S. Buttignol-S. Confente, 10072 Mappano di Caselle, Italy; simona.butti@tin.it

**Keywords:** obesity, family-centred approach, caregiver, compliance, nutrition

## Abstract

Obesity represents a one of the most significant healthcare issues facing human and companion animal populations worldwide. A complex relationship commonly exists between owners and their companion animal, particularly around feeding behaviour. Obese companion animals commonly live alongside caregivers who are also struggling with their own body weight. This case report highlights the importance of a *family-centred* approach to canine obesity as a way to engage with the pet’s caregivers to help maximize their compliance towards the successful implementation of a tailored weight loss programme. *Lara*, an overweight dog weighing 35 kilos with a body condition score (BCS) of 7–7.5/9, was referred for a nutritional assessment. A comprehensive, pro-active and multidisciplinary protocol, tailored towards a family-centred approach, was established. After a 16-week programme, *Lara* reached the target body weight. The caregivers’ compliance was assessed as being excellent; they also reassessed their individual lifestyle and were able to increase awareness towards their own nutritional issues and body weight perception, resulting in weight loss in all caregivers. *Lara*’s case report represents how a family-centred approach can lead to successful patient weight loss and to a modification in the caregivers’ way of thinking about nutrition and their own lifestyle, with the final goal of living a healthier and longer life together.

## 1. Introduction

Human and companion animal obesity represents a significant healthcare issue facing both populations and is of increasing concern, particularly in developed countries [1,2]. A complex relationship commonly exists between owners and their companion animals, particularly around feeding behaviour [3,4]. Obese pets are commonly kept by humans who are struggling with their own body weight and share the same environment, feeding habits and risks to health of being overweight [5,6,7,8]. Obesity has been defined as “*an accumulation of excessive energy storage in the form of adipose tissue, sufficient to contribute to disease*” [8,9]. Dogs are judged to have an ideal body composition with 15 to 20% body fat and can be classed as *overweight* when their relative body weight is between 10 and 20% above their ideal weight and *obese* when this figure is above 20% [8]. Large studies in Great Britain and the United States indicate that the prevalence of overweight and obese dogs is between 24 and 30% [10,11], with a higher risk in middle-aged and neutered animals [11,12]. Although it is widely accepted that being overweight negatively influences health, life-span and quality of life [13,14], the majority of owners tend to underestimate the problem [15,16] without recognising this condition as an illness. Furthermore, veterinarians often struggle in establishing an effective interaction and communication with clients when dealing with nutritional issues, especially with respect to obesity [17,18]. Additionally, the veterinary healthcare team often finds it difficult to convince owners to adhere to the weight reduction programme for obese pets; “*Many veterinary healthcare teams are reluctant to tell a client that their pet is obese*” [19]. Several risk factors have been proposed in dogs as the origin of excessive adiposity which characterises the disease, including genetics, neutering, decreased activity level, high fat/high energy diets and gut microbiota [4,5,20]. A recent publication found no effect of neutering on voluntary food intake or the satiety state, but it did reduce activity levels in comparison to control dogs [21]. Nevertheless, all significant causes for developing excessive adiposity and subsequent comorbidities are still to be fully elucidated [4,5]. For these reasons a multi-modal, pro-active medical approach to the obese companion animal is warranted in order to guarantee long-lasting compliance and successful weight loss. One strategy central to success is client communication [2,19,22], which becomes even more critical if the owner is also overweight [2,19]. The aim of this case report is to highlight the importance of a *family-centred* approach to obesity in a pet dog as a way to ensure all caregivers are compliant towards their pet’s tailored weight loss programme.

## 2. Signalment, History and Assessments

*Lara*, a 4-year old neutered female, large-sized mixed-breed dog weighing 35 kilos, was referred to the Department of Veterinary Science, Turin University (IT) by her general practitioner (S.B) in order to receive a nutritional assessment. SB’s major concern was *Lara’s* progressive weight gain that had occurred since her ovariectomy procedure about 1 year before referral. The referring vet had already prescribed *Lara* with several commercial weight-reducing diets without obtaining a significant and stable decrease in body weight and body fat. This lack of success tended to affect the caregivers’ motivation, which led to reluctance to strictly adhere to an established nutritional protocol and failure to complete dietary trials. 

*Lara* was admitted to our facility for a complete nutritional assessment. The first consultation was carried out in accordance with WSAVA guidelines [23] and was composed of three phases: Phase 1: patient assessment (signalment, medical history, physical examination, laboratory testing); Phase 2: nutritional assessment; Phase 3: owner interview. 

Routine laboratory evaluation, including a complete blood count, serum biochemistry and urinalysis, were performed in order to further evaluate the health status of the patient and to establish reference values for *Lara* before starting the weight loss programme. Evaluation of *Lara’s* thyroid function was also advised to the owners, as it was important to know thyroid status as a possible differential diagnoses causing a predisposition to weight gain and lethargy. Evaluation of serum inflammatory cytokines and redox balance parameters was also suggested, but were declined due to economic restraints.

## 3. Results

**Figure 1:**
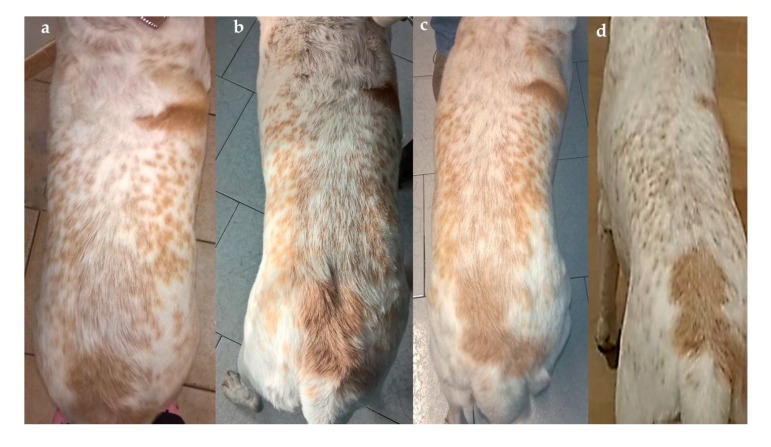
*Lara’s* dorsal view (non-standardized picture, according to Gant and others, 2016 [24], at admission (**a**), and after 4 (**b**), 10 (**c**) and 16 (**d**) weeks from the beginning of the weight loss programme. Despite the non-standardized method of photography, it is clear how the body condition score (BCS) and body shape changed over time. Notice the absence of a waistline and the fat deposit over the lower back in Figure 1**a** (BCS: 7–7.5/9) and then the visible waist in Figure 1**d** (BCS: 5/9). The scoring system was: ideal weight (BCS: 4–5/9), overweight (BCS: 6–7/9), and obese (BCS: 8–9/9) [24].

**Figure 2:**
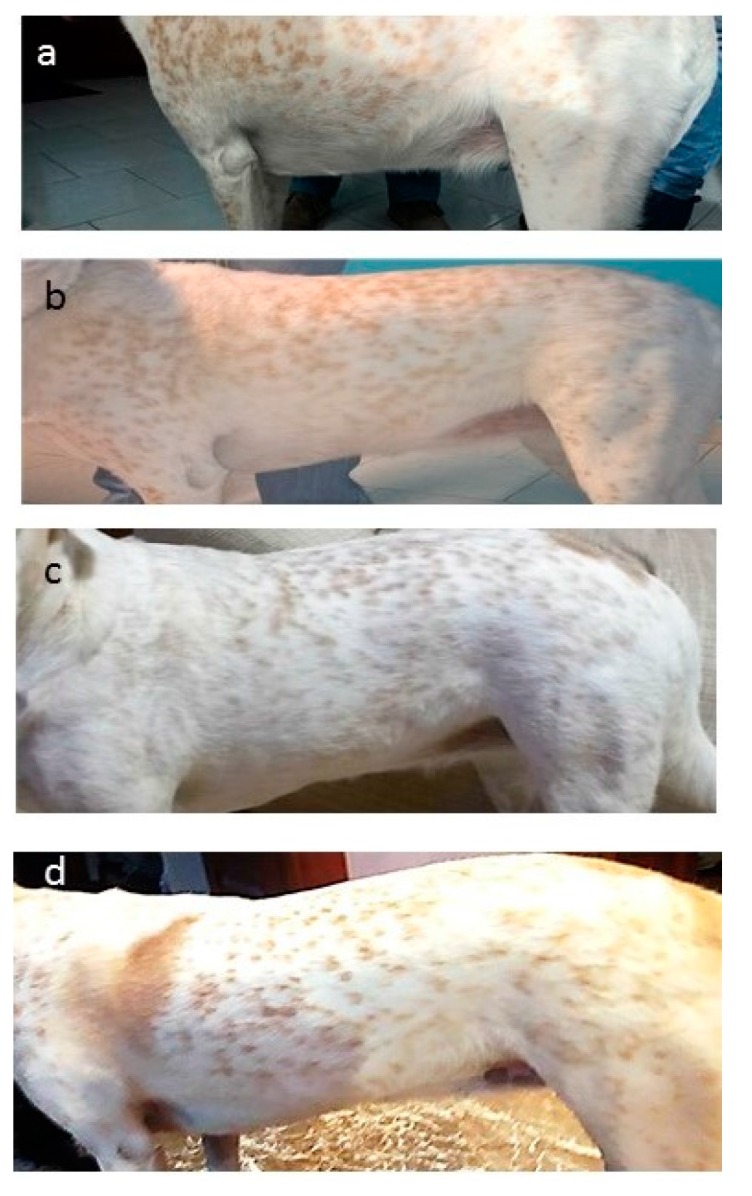
*Lara’s* left lateral view (non-standardized picture, according to Gant and others, 2016 [24], at admission (**a**), and after 4 (**b**), 10 (**c**) and 16 (**d**) weeks from the beginning of the weight loss programme. As with the dorsal photography it is clear how BCS and body shape changed over time. Notice the abdominal distension in Figure 2**a** (BCS: 7–7.5/9) and lack of an abdominal tuck, then the appearance of an abdominal tuck in Figure 2**d** (BCS: 5/9).

From Phase 1 it emerged that *Lara* was a shy but alert dog who had developed a certain laziness and reluctance to walk during the last year. Her physical examination was unremarkable, with the exception of an overweight body condition score (BCS) of 7–7.5 out of 9 [24], with a muscle mass index (MMI) still within the normal range (Figure 1a and Figure 2a). 

No orthopedic, respiratory or cardiovascular abnormalities were found as possible aetiologies for the reported lethargy. Findings recorded during this phase are shown in Table 1. 

Phase 2 was focused on obtaining a complete dietary history. Apart from periods in which a commercial weight-reducing diet was intermittently administered, *Lara* was fed with approximatively 300–350 g of a dry basic pet food (kcal metabolic energy (ME)/100 g ≈ 364) and 350 g of ground beef per day, divided into one or two meals. Extras, such as slices of bread or bread-sticks, were also routinely added to the diet by the owner and dog treats were occasionally dispensed by other family members. A daily caloric intake of ≈1500–1600 kcal ME was estimated. 

Phase 3 was focused on the caregivers’ attitude towards *Lara’s* nutrition and management to identify critical points and weaknesses which might have played a role in previous unsuccessful attempts of weight loss. From the interview it clearly emerged that one of the caregivers’ major concerns was that *Lara* would suffer from starvation during the weight loss programme, begging for extra food all day long. Another key point that became obvious was the distorted perception of wellness they had towards *Lara*, as they saw her round body shape as a direct consequence of being happy and healthy. Furthermore, *Lara* was rarely walked outside; the belief was that having free access to a private yard would provide her with enough motivation to perform sufficient physical exercise and burn off extra calories. Lastly, all the caregivers were struggling with the maintenance of their own lean body weight and were used to eating extremely processed, high-calorie food and living an inactive lifestyle. 

Results of the complete blood count, serum biochemistry and urinalysis are shown in Table 2.

## 4. Diagnosis, Treatment and Follow-Up

A diagnosis of primary obesity was established taking the results from the patient and nutritional assessment, owner interview and laboratory evaluation. Evaluation of the BCS, MMI and morphometric measurements estimated the total body fat to be 33–35%. The excess of adiposity was mainly attributed to the caregivers’ approach to overfeeding calories (including treats), a lack of physical exercise and being neutered. A genetic predisposition could also not be excluded. 

A personalized weight loss programme was designed, according to Toll’s guidelines [8].

The protocol was focused on three areas:

(1) Reducing *Lara’s* daily caloric intake while promoting satiety. This was done by prescribing a low-energy density, high-protein and L-carnitine-supplemented commercial weight loss diet [25] (Table 3).
(2) Implementing physical activity by scheduling a daily-based work-out.
(3) Directly involving all caregivers through communicating a family-centred approach targeted towards monitoring the effectiveness of the weight loss programme, and increasing their awareness on how treats and any extra calories would negatively influence the outcome.

The ideal body weight (IBW) was calculated using the following equation [8]:

Ideal body weight = current weight × (100 – percent body fat [%BF])/0.80

An IBW of 28.4–29.3 kg was obtained. A realistic IBW of 29.5 kg was set.

Total weight reduction of 5.5 kg was required, with a 1% per week rate of weight loss (0.35 kg/week) set as the desired target. A maximum weight loss rate of 2% per week (0.7 kg/week) would be tolerated. An ideal (minimum) time interval of 16 weeks at 1% (8 weeks at 2%) was calculated in order to reach the IBW of 29.5 kg.

The resting energy requirement (RER) at IBW was estimated by using the following equation: [8]:

RER IBW = 70 × (29.5)^0.75^

*Lara’s* RER at IBW was 886 kcals and the daily energy requirement (DER) was 1.4× RER at IBW or 1240 kcals. *Lara’s* typical daily caloric intake of ≈1500–1600 kcals was therefore much higher than the estimated DER. A daily energy intake of 843 kcals (68% of IBW DER) was calculated for the desired 1% rate of weight loss.

A mixed-feeding regimen was prescribed (50% dry food + 50% canned food) by using a commercial product [25].

The selected food provided 333.4 kcal/100 g (dry) and 100.9 kcal/100 g (canned). Therefore, 125 g/day of dry food and 420 g/day of canned food (corresponding approximatively to 1 tin) were suggested, divided into three equal meals/day. One medium apple/day and/or a small amount of green vegetable (i.e., spinach, zucchini, green beans) were tolerated as extras or treats. 

A 30-min walk was advised every day for the first two weeks of the programme. Then, an extra 10-min per walk was added weekly, until a 60-min daily work-up was reached.

The owner was trained to monitor *Lara’s* improvements by using the “WALTHAM S.H.A.P.E.^TM^ (Size, Health And Physical Evaluation) system for Dogs” [26] and record the result every four weeks (Table 4). The concept on which this guide is based is that it consists of an algorithm where a series of consecutive questions, linked to a simple physical examination of the dog’s body composition, are asked of the owner, in a simple and clear manner. Only a “Yes” or “No” reply is accepted. Depending on each response a further series of questions are asked, eventually leading to a final score which is directly related to the dog’s body composition. Scoring ranges from extremely thin to severely overweight; score A through to G, respectively. The explanation of each score is provided in the legend of Table 4. 

The aforementioned system was useful to engage with the caregivers and highlight how they had tended to underestimate *Lara’s* body condition score, as was recorded in the first consultation, and was used to help reinforce the need for dietary intervention.

An exercise and calorie-counting mobile application (SlimDoggy App, AppStore) was also given in order to track dog’s daily activities, treats received and calories burned/eaten. 

A standardized quality of life (QoL) questionnaire, formulated ad hoc by adapting QoL indicators already published [27], was also given to all *Lara*´s caregivers, to be filled in at the beginning and at the end of the 16-week weight loss programme (Table 5). Briefly, the questionnaire comprised of five simple descriptor items (*levels of spontaneous physical activity, happiness, vitality, anxiety and food request*), scored on a 0–6 scale and directly related to emotional, physical and overall wellness. As *Lara* was known to be a shy dog, the questionnaire was tailored to provide evidence not only related to her physical status but also to her behavioural response during the 16-week programme.

**Figure 3:**
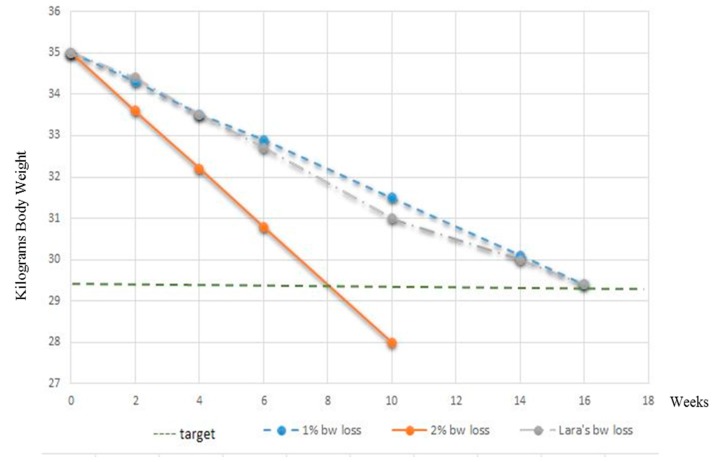
Plot of *Lara’s* body weight loss from the start of, and during the full 16 week programme.

Follow-up consultations were scheduled after 2, 4, 6, 10, 14 and 16 weeks. Follow-up phone calls were established after 1, 3, 5, 8 and 12 weeks. During each recheck, elements from Phase 1, Phase 2 and Phase 3 were briefly reassessed and recorded (Table 1). *Lara’s* lateral and dorsal body profiles were photographed on admission and after 4, 10 and 16 weeks from the beginning of the weight loss programme. Data tracked by the SlimDoggy App were analysed. Complete blood work and urinalysis were repeated at the end of the 16-week programme. QoL questionnaires were evaluated during the last week of the weight loss programme. The plot of *Lara’s* body weight loss during the 16-week programme is seen in Figure 3.

The reduction in body weight obtained (grey line) was in agreement with the desired rate of 1% per week weight loss (blue line) during each clinical recheck. No changes in daily energy intake were therefore made during the 16-week weight loss programme. *Lara’s* physical examinations were within normal limits at each follow-up consultation and changes in her body shape and percentage of body fat soon became apparent, as seen in Figure 1a–d and Figure 2a–d and Table 4. Importantly, the caregivers’ compliance and motivation was excellent during the whole period; the use of the mobile application and the request to monitor improvements with the WALTHAM S.H.A.P.E.^TM^ system contributed greatly to the optimal weight loss achieved. All caregivers also reported that the dog had an improved QoL (Table 5), with an increased playfulness and a positive attitude toward physical exercise. 

Lethargy and laziness were not reported anymore. Requests for extra food, apart from regular feeding times, diminished. Satiety was satisfactorily controlled even with a diet relatively low in crude fibre (2.3% dry matter [dry formula [25]]). Once the ideal body weight of 29.5 kg was reached a feeding plan for weight-maintenance was arranged. *Lara* was switched to a calorie-restricted food [28] and fed for maintenance of the ideal body weight. Obese pet dogs that have completed weight loss programmes can have an average daily metabolizable energy intake of 285 kJ (68 kcal)/kg^0.75^/day [29] which is considerably lower than the current maintenance energy requirement recommendation for inactive adult pet dogs of 398–440 kJ (95–105 kcal)/kg^0.75^/day [30]. Therefore, long-term feeding of a calorie-restricted low-fat food after weight loss can significantly limit regain in the follow-up period, likely by limiting calorie intake [31], *Lara* was now only given 10% more calories than she was fed for weight loss. A transition period of a few days to the new weight-maintenance food was advised. Daily physical exercise was also encouraged during this phase. Rechecks were scheduled after 2, 6 weeks and then every 3 months.

At the time of writing, 3 months have passed from the beginning of weight-maintenance protocol. *Lara* now weighs 29.3 kg, has a BCS of 5 out of 9 and an estimated percentage of total body fat of 15–17%. She is clinically healthy, happy and playful. Additionally, all the caregivers reassessed their own lifestyle, electing to adopt a more active one (which even included a gymnasium membership for one family member), and they also increased their awareness about nutritional issues by moving away from eating highly processed foods and towards adopting a healthier diet. At the end of the 16-week weight loss programme of *Lara* the lead clinician (A.C) was informed that her three caregivers had lost a combined total of 8 kg of body weight between all of them.

## 5. Discussion

Obesity is the most important nutritional issue confronting the human population in industrialized countries [32]. In dogs the prevalence of obesity has grown in last decades and it mirrors the increase in the human population [1,2,19]. Similar environmental factors shared by both species, such as inadequate exercise and overfeeding are all implicated aetiologies, as well as a genetic component [4,33] and in this case report, all the aforementioned risks potentially affecting *Lara's* weight have been present in her medical history. The increase in total body fat content, however, represents not only a cosmetic or social issue, but mainly a medical problem [34]. The metabolic effects of obesity on insulin resistance and development of hyperlipidaemia, the mechanical stress of extra weight on musculoskeletal system, and consequences of the chronic inflammatory condition related to increased adiposity are well established in literature [34,35]. Fortunately, laboratory analysis performed on *Lara* did not reveal biochemical abnormalities or signs of insulin resistance. This can be explained by the relatively young age of the patient and the moderate stage of obesity which characterized *Lara* (33–35% of total body fat). However, markers of chronic inflammation and a complete endocrine profile were not performed due to the owners’ economic restraints. Thus, a chronic inflammatory condition or a metabolic imbalance could not be completely excluded in this patient. In support of this hypothesis, we could assume that Lara’s lethargic behaviour and her reluctance to walk already represented the consequence of the mechanical stress that the excessive amount of fat exerted on the musculoskeletal system, as well as her neutering status [21]. The quality of life questionnaire charted key indicators that helped further involve the family in the assessment of their pet´s overall well-being, both at the start and at the end of the 16-week programme. As *Lara* was noted to be a shy dog, including a *vitality* assessment helped demonstrate to her caregivers the positive improvement in her wellbeing following weight loss. Increased physical activity, mental alertness, and extroversion all contribute to vitality [27]. The improvement in this indicator after 16 weeks would help to reduce the risk of recidivism towards weight gain due to the caregivers returning to their original feeding and lifestyle approach with *Lara*.

Despite the potential associated co-morbidities, overweight and obesity still remain frustratingly difficult conditions to address and reverse [19] and acknowledgment of the clinical consequences of obesity, by both the veterinarian and the owner, is critical to effectively address the problem [2,19,22]. Furthermore, in order to achieve successful weight loss, all the patient’s caregivers must alter often long-standing feeding practices and habits [4,19]. To change these traits, a clear understanding of the benefits of reaching and maintaining a lean body condition is desirable. Several studies have shown that owners tend to underestimate their pets’ body condition [16,36] and consider their habits in overfeeding the dog, by giving extras and treats as a way to reinforce the human–animal emotional bond [3,36]. In addition, some owners assert that they believe food restriction would cause their pet to suffer and that they instead would prefer their pet to be “happy” even if they are overweight. This was exactly what emerged from *Lara’s* owners’ interview and from the analysis of the WALTHAM S.H.A.P.E.^TM^ system’s results. 

In humans there is great interest in addressing childhood obesity by moving parents from indulgent parenting, where parents may use food to soothe or control behaviour, towards responsive parenting which emphasizes responsive feeding guidance by “*helping parents learn to identify and respond sensitively to infant hunger-satiety cues and to recognize and respond to other distresses including the infant’s need for sleep*” [4]. A prospective, randomized controlled trial (INSIGHT project; Intervention Nurses Start Infants Growing on Healthy Trajectories) is evaluating if a responsive parenting intervention does indeed prevent rapid infant weight gain and childhood obesity among first-born infants [37]. It is feasible that principles from INSIGHT could be adapted (e.g., early feeding practices and responsive ownership) to companion animals [4]. 

Another key-factor that has to be considered when dealing with an obese patient is client communication [2,19]. Since the WSAVA Nutritional Assessment Guidelines were first published [23], nutritional counselling has been considered as a minimum standard of veterinary care, to be included in every patient interaction [6]. Nutritional assessment has to be considered as an iterative process that encompasses evaluation of the patient, the food being offered and consumed, the dog’s environment and the feeding management strategy being used by the caregivers [38]. In order to succeed, however, the development of strong communication skills and a pro-active, non-judging attitude is mandatory; “*All members of the healthcare team should be trained in effective communication in order to maximize the success of a weight reduction and maintenance programme and achieve client satisfaction*” [19]. 

A clear message came to light from the recent congress “Animal Obesity––causes, consequences and comparative aspects” [33], held at the Swedish University of Agricultural Sciences, where researchers with a common interest in different issues related to animal obesity established national and international contacts. The take-home message was: *only a holistic and multidisciplinary approach to the obese patient could be able to influence owner’s understanding of healthy nutrition and lifestyle and commitment to change* [33]. Invigorated by that message, great importance was given to all the caregivers’ emotions and expectations when dealing with *Lara’s* case by carefully listening and addressing their doubts and false beliefs. Furthermore, in order to reinforce all caregivers’ commitment to change their nutritional and lifestyle habits, practical tools (such as the WALTHAM S.H.A.P.E.^TM^ system, WALTHAM Centre for Pet Nutrition, Leicestershire, UK), QoL questionnaires (Alessia Candellone, Department of Veterinary Science, University of Turin, Grugliasco, Italy) and the SlimDoggy App. (Petnet, Los Angeles, CA, USA: http://slimdoggy.com/) were provided as a way to directly involve them in the monitoring process. 

The positive response and long-term outcome of all of *Lara’s* caregivers towards their own health and lifestyle was an endorsement of the family-centred approach to this case. Encouraging regular joint exercise is a simple and effective way of increasing the owner–pet bond as well as facilitating weight loss. A study of obese caregivers who undertook modest regular exercise with their obese dog showed a positive benefit for both species [39]. Over the 12 months the time spent by the caregiver in physical activity (hours/week) increased, with 66% of this time being with their dog, and the mean weight losses in this period were 4.7% for owners and 15% for the dogs. The authors concluded that “*Consideration of social support for weight loss of family members, friends, and coworkers should be extended to include pets*” which helps endorse a One Health approach to addressing obesity [2,4,19]. While this case was not based on a One Health approach per se [2,4,19] (all caregivers’ body weight parameters, lifestyle and eating habits were not formally recorded), it does however give tangible support to the importance of effective communication, of involving all caregivers in decisions and case management protocols, and providing regular follow-up communication to help reinforce the veterinarian–client bond. The improvement in the weight, eating habits and exercise routines of all the caregivers was a welcomed additional reward for the time spent communicating on their pet’s weight loss programme, communication undertaken by a single veterinary healthcare professional. Veterinarians have a role in improving the health of pets, their owners and all caregivers [2] and the One Health approach to obesity requires both veterinary and medical healthcare professionals to be involved in interdisciplinary collaboration towards the companion animal and owner, endorsing more effective communication with patients/pet owners [2,4,19]; “*The technique of “client-centred communication” provides motivation for healthy habits. Small and sustainable changes in lifestyle can lead to long-term success*” [2].

## 6. Conclusions

*Lara’s* case report represents an example of how a comprehensive, pro-active and multidisciplinary approach can lead to successful weight loss and to a positive modification in all the caregivers when thinking about their own nutrition, with the final goal of living a healthier and longer life together. One clear conclusion from this report was that as veterinarians we need to be even more family (client)-centred in our communication, adopting a One Health approach to weight loss, weight management, healthy lifestyle and healthy eating habits.

## Figures and Tables

**Table 1:** Main findings recorded during Phase 1 (patient assessment) at the admission and after 2, 4, 6, 10, 14 and 16 weeks.

Patient Assessment	At Admission	After 2 Weeks	After 4 Weeks	After 6 Weeks	After 10 Weeks		After 14 Weeks	After 16 Weeks
Mentation	QAR	QAR	BAR	BAR	BAR		BAR	BAR
Weight (kg)	35	34.4	33.5	32.7	31		30	29.5
BCS (1–9)	7–7.5	7	6.5	6	5.5		5.5	5.0
MMI	wnl	wnl	wnl	wnl	wnl		wnl	wnl
Morphometric measurements (cm):								
“Hock to stifle” (HS)	24.5	-	-	-	-		-	-
Pelvic circumference (cms)	76	74	71.8	70.2	69.5		68	67.3
Rectal temperature (Celsius degree)	38.6	38.3	38.8	38.7	38.3		38.5	38.6
RR (bpm)	26	24	25	22	24		22	20
HR (bpm)	82	80	79	84	80		81	80
CRT (s)	<2	<2	<2	<2	<2		<2	<2
Mucous membranes	pink	pink	pink	pink	pink		pink	pink
Hydration	adequate	adequate	adequate	adequate	adequate		adequate	adequate
Peripheral lymph nodes	wnl	wnl	wnl	wnl	wnl		wnl	wnl
CV	wnl	wnl	wnl	wnl	wnl		wnl	wnl
EENT	wnl	wnl	wnl	wnl	wnl		wnl	wnl
GI	wnl	wnl	wnl	wnl	wnl		wnl	wnl
GU	wnl	wnl	wnl	wnl	wnl		wnl	wnl
INTEG	wnl, with the exception of a focal exfoliative dermatitis in the back region	wnl. No signs of dermatitis	wnl	wnl	wnl		wnl	wnl
M/S	Lethargy and reluctance to walk reported by the owner. Occasional stiffness, especially while climbing stairs. No musculo-skeletal abnormalities detected during physical examination	Lethargy and reluctance to walk still reported, but reduced in severity and frequency	Increased mobility. Climbing stairs became easier.	No problem in climbing stairs. The dog is more active and playful	No problem in climbing stairs. The dog is more active		No problem in climbing stairs. The dog is more active and playful	No problem in climbing stairs. The dog is more active and playful
NERV	wnl	wnl	wnl	wnl	wnl	wnl	wnl	wnl
RESP	wnl	wnl	wnl	wnl	wnl	wnl	wnl	wnl

Legend: BAR: bright, alert and responsive; BCS: body Condition Score; CRT (seconds): capillary refill time; CV: cardiovascular; EENT: eyes, ears, nose, throat (and mouth); GI: gastrointestinal; GU: genitourinary; HR (beats per minute): heart rate; INTEG: integument; MMI: muscle mass index; M/S: musculoskeletal; NERV: nervous system; QAR: quiet, alert and responsive; RESP: respiratory; RR (breaths per minute): respiratory rate; wnl: within normal limits.

**Table 2:** *Lara’s* haemato-biochemical results, urinalysis and thyroid function at admission and after 16 weeks from the beginning of the weight loss programme.

Haemato-Biochemical Parameters	At Admission	After 16 Weeks	Reference Range
WBC (×10^3^/μL)	16.2	14.3	6.0–17.0
RBC (×10^6^/μL)	6.5	6.8	5.5–8.5
HGB (g/dL)	13.7	14.2	11.0–19.0
HCT (%)	46	47	39.0–56.0
PLT(×10^3^/μL)	311	386	117–460
BUN (mg/dL)	22	23	7–25
CREA (mg/dL)	1.1	1.0	0.3–1.4
Total bilirubin (mg/dL)	0.3	0.2	0.1–0.6
Total proteins (g/dL)	7.3	7.7	5.4–8.2
Albumin (g/dL)	3.8	3.9	2.5–4.4
Globulin (g/dL)	3.5	3.8	2.3–5.2
ALT (IU/L)	46	39	10–118
ALP (IU/L)	24	32	20–150
Cholesterol (mg/dL)	350	200	130–370
Triglyceride (mg/dL)	98	57	10–100
Calcium (mg/dL)	10.9	10.8	8.6–11.8
Phosphorus (mg/dL)	5.2	4.7	2.9–6.6
Sodium (mEq/L)	147	152	138–160
Potassium (mEq/L)	5.6	5.0	3.7–5.8
Urinalysis *	**At admission**	**After 16 weeks**	**Reference range**
pH	6.5	6.0	5.5–7.5
Specific gravity	1.035	1.045	1.001–1.050
Colour	Amber yellow	Amber yellow	/
Turbidity	Transparent	Transparent	/
Protein	Traces	Absent	≤10 mg/dL
Glucose	Negative	Negative	Negative
Ketones	Negative	Negative	Negative
Sediment (Casts, WBC, RBC, cells, crystals, bacteria)	Unremarkable, except from Cocci/few (due to contamination at collection)	Unremarkable	/
**Thyroid function**	**At admission**	**After 16 weeks**	**Reference range**
Free T4 (pmol/L)	27	33	15–45
Total T4 (µg/dL)	1.8	2.1	1.0–4.0
TSH (ng/mL)	<0.5	Not performed	<0.5

* urine collected by spontaneous micturition; WBC: White Blood Cells; RBC: Red Blood Cells; HGB: Haemoglobin; HCT: Haematocrit; PLT: Platelets; BUN: Blood Urea Nitrogen; CREA: Creatinine; ALT: Alanine aminotransferase; ALP: Alkaline phosphatase .

**Table 3:** Nutrient composition of Eukanuba Veterinary Diets Restricted Calorie, dry and canned food [25]. All values are “as-fed” except those indicated with an asterix *.

Nutrient	Canine Dry Food	Canine Canned Food
Water	8.00%	77.00%
Protein	28.80%	8.00%
Fat content	8.00%	4.50%
Omega-6 fatty acids	1.50%	0.62%
Omega-3 fatty acids	0.20%	0.12%
Crude ash	6.70%	1.60%
Crude Fibres	2.10%	0.50%
Calcium	1.20%	0.25%
Phosphorus	1.00%	0.20%
Vitamin A	70,000 IU/kg	18,000 IU/kg
Vitamin D3	900 IU/kg	300 IU/kg
Vitamin E (α-tocopherol)	100 mg/kg	50 mg/kg
L-carnitine	50 mg/kg	10 mg/kg
Glucosamine *	475 mg/kg	-
Chondroitin sulphate *	45 mg/kg	-
Fibre source	Beet pulp	Beet pulp
Energy Kcal/Kg (calculated with modified Atwater)	3334	1009

* level added to the formulation pre-manufacturing.

**Table 4:** Results of the WALTHAM S.H.A.P.E.^TM^ system, recorded by the owners every four weeks.

	After 4 Weeks	After 8 Weeks	After 12 Weeks	After 16 Weeks
Results of WALTHAM S.H.A.P.E.^TM^	Score F	Score E	Score D	Score D

Score A: extremely thin: Score B: thin; Score C: lean; Score D: dog with an ideal weight; Score E: mildly overweight; Score F: moderately overweight; Score G: severely overweight.

**Table 5:** Results of the quality of life (QoL) questionnaire formulated ad hoc, completed by the owners at the beginning and at the end of the weight loss programme.

Descriptor Items	At the Beginning of the Weight Loss Programme	At the End of the 16-Week Weight Loss Programme	Reference Range
Level of spontaneous physical activity, directly related to physical wellness	3	5	0–6
Level of happiness, directly related to emotional wellness	4	6	0–6
Level of vitality, directly related to overall wellness	2	5	0–6
Level of anxiety, directly related to emotional disturbance	5	5	0–6
Level of food request, directly related to hunger and satiety	6	4	0–6

The higher the score the greater the descriptor items were being expressed, as evaluated by all caregivers.
